# Causal effects of serum calcium, phosphate, and 25-hydroxyvitamin D on kidney function: a genetic correlation, pleiotropic analysis, and Mendelian randomization study

**DOI:** 10.3389/fendo.2024.1348854

**Published:** 2024-09-30

**Authors:** Yanjun Liang, Shuang Liang, Dayang Xie, Xinru Guo, Chen Yang, Tuo Xiao, Kaiting Zhuang, Yongxing Xu, Yong Wang, Bin Wang, Zhou Zhang, Xiangmei Chen, Yizhi Chen, Guangyan Cai

**Affiliations:** ^1^ Medical School of Chinese People’s Liberation Army (PLA), Beijing, China; ^2^ Department of Nephrology, First Medical Center of Chinese People’s Liberation Army (PLA) General Hospital, Nephrology Institute of the Chinese People’s Liberation Army, State Key Laboratory of Kidney Diseases, National Clinical Research Center for Kidney Diseases, Beijing Key Laboratory of Kidney Disease Research, Beijing, China; ^3^ Department of Nephrology, Characteristic Medical Center of Chinese People’s Armed Police Force, Tianjin, China; ^4^ School of Medicine, Nankai University, Tianjin, China; ^5^ Department of Nephrology, Hainan Hospital of Chinese Chinese People’s Liberation Army (PLA) General Hospital, Academician Chen Xiangmei of Hainan Province Kidney Diseases Research Team Innovation Center, Sanya, China; ^6^ The Second School of Clinical Medicine, Southern Medical University, Guangzhou, China

**Keywords:** calcium, phosphate, 25-hydroxyvitamin D, parathyroid hormone, kidney function, Mendelian randomization, genetic correlation, pleiotropic analysis

## Abstract

**Background:**

Existing studies investigating the impact of serum calcium (Ca), phosphate (P), 25 hydroxyvitamin D (25[OH]D), and parathyroid hormone (PTH) levels on kidney function have produced inconsistent results. Further research is needed to establish the direct causal relationship between these factors and kidney function.

**Methods:**

The study used genome-wide association study datasets for exposure and outcome, mainly derived from the UK Biobank and CKDGen Consortium, with sample sizes ranging from 3,310 to 480,699 individuals of European ancestry. Heritability and genetic correlations among these phenotypes were assessed using linkage disequilibrium score regression (LDSC) and phenotypes with a heritability z-score <4 were excluded from further analyses. Pleiotropic analyses were performed to identify potential horizontal pleiotropic variants at gene and LD-independent locus levels. Mendelian randomization (MR) analysis, using instrumental variables (IVs) based on two distinct selection criteria, was conducted to investigate the potential causal relationships between serum Ca, P, 25(OH)D, PTH, and kidney function.

**Results:**

PTH was excluded from further analysis due to a heritability z-score < 4. Genetic correlations were observed between serum Ca and urine albumin-to-creatinine ratio (UACR) (rg = 0.202, P-value = 5.0E−04), between serum 25(OH)D and estimated glomerular filtration rate using serum creatinine (eGFRcrea) (rg = -0.094; P-value = 1.4E−05), and between serum 25(OH)D and blood urea nitrogen (BUN) (rg = 0.127; P-value = 1.7E−06). In univariable MR analysis using IVs based on two different selection criteria, it consistently demonstrated that genetically predicted serum Ca consistently showed an increase in UACR (beta 0.11, *P-*value 2.0E−03; beta 0.13, *P-*value 2.0E−04). Similarly, serum P was associated with a decrease in eGFRcrea (beta −0.01, *P-*value 2.0E−04; beta −0.005, *P-*value 2.0E−03) and an increase in BUN (beta 0.02, *P-*value 3.0E−03; beta 0.02, *P-*value 7.5E−07). The influence of serum P on kidney function was further supported in multivariable MR analysis. However, genetically predicted 25(OH)D did not have a significant impact on kidney function.

**Conclusions:**

Elevated serum Ca or P levels could both impair kidney function, whereas 25(OH)D has no impact on renal function.

## Introduction

Chronic kidney disease (CKD) is a significant contributor to the global burden of non-communicable diseases. Its common complications, including calcium (Ca) and phosphate (P) metabolic disorders, 25-hydroxyvitamin D (25[OH]D) deficiency, and hyperparathyroidism, worsen with the decline of renal function. Several clinical studies have focused on the potential impact of these complications on kidney function. However, the results were inconsistent, which may mainly be due to the interference of confounders or mediators and the uncertainty regarding the occurrence of the exposure factors, i.e., whether these occurred before the outcome.

For instance, a retrospective cohort study provided evidence that higher serum Ca levels were associated with a reduced risk of kidney failure (1). Conversely, another study indicated that increased Ca intake led to elevated serum Ca and creatinine levels, irrespective of random vitamin D intake (2). Moreover, in individuals with hypoparathyroidism, it was observed that prolonged hypercalcemia resulting from vitamin D and Ca supplementation was linked to a decline in renal function (3). Similar inconsistencies have been observed in studies investigating the impact of serum P levels on kidney function (1, 4–6).

In terms of parathyroid hormone (PTH) and its role in kidney function, a retrospective cohort analysis found that PTH levels>50 pg/mL in patients with CKD 3−4 were associated with an escalating combined risk of death or renal replacement therapy (7). Furthermore, a Phase 3 PaTHway Trial examined the efficacy and safety of TransCon PTH replacement therapy in individuals with hypoparathyroidism. The study findings revealed that participants receiving TransCon PTH experienced an increase in mean estimated glomerular filtration rate using serum creatinine (eGFRcrea) from 67.5 to 75.6 mL min^−1^ per 1.73 m^2^, whereas no significant change in eGFRcrea was observed in the placebo group. However, it was important to note that other factors influenced by medication administration complicated the assessment of the protective effect of PTH on renal function, which fell beyond the scope of this trial (8).

Randomized controlled trial (RCT), which was widely regarded as the gold standard for establishing causation, could still yield false positive or false negative results due to limitations in sample size, duration of follow-up, and potential interference from unknown confounders. For instance, an RCT involving 61 CKD stage 3–4 patients found that oral vitamin D analogs administered over 6 months reduced urinary protein levels, irrespective of renin–angiotensin system inhibitor use (9). However, another RCT with 43 CKD stage 3–4 patients, using similar treatments and follow-up duration, failed to support these findings (10). Additionally, a meta-analysis suggested that the administration of vitamin D receptor agonists decreased the eGFRcrea (11).

To address the limitations of clinical studies, a combination of genetic analyses can be employed. Linkage disequilibrium score (LDSC) regression allows for the assessment of single-nucleotide variant (SNV)-based phenotype heritability and co-heritability between two traits, playing a pivotal role in providing valuable etiological insights and aiding in the prioritization of potential causal relationships (12). Pleiotropy refers to the phenomenon of one mutation affecting multiple traits. This includes vertical pleiotropy, where one variant influences both traits in succession, reflecting the causal relationship between the two traits, and horizontal pleiotropy, independently influencing the two traits, merely reflecting the correlation between the two traits. Pleiotropy analyses quantify horizontal pleiotropic relationships at gene and LD-independent locus levels, aiding in the detection of potential horizontal pleiotropic variants to prevent false positives in subsequent Mendelian randomization (MR) analyses. MR analysis refers to an analytic approach for assessing the causality of an observed association between a modifiable exposure and a clinically relevant outcome, focusing on the vertical pleiotropy relationship. Because MR exploits the fact that genotypes are not generally susceptible to reverse causation and confounding due to their fixed nature and Mendel’s first and second laws of inheritance (13). Univariable MR estimates the total causal effect of the exposure on the outcome. On the other hand, multivariable MR extends the analysis to consider a set of potentially correlated candidate exposure factors (>1). It evaluates the direct causal effect of each exposure factor on the outcome while accounting for the other exposure factors (14).

To our knowledge, there have been univariable MR studies that have found no effect of 25(OH)D on kidney function (15). However, there is a noticeable research gap in the literature when it comes to MR studies investigating the impact of Ca, P, and PTH on kidney function, as well as the use of multivariable MR to estimate the causal effects of Ca, P, PTH, and 25(OH)D on kidney function. Our research aims to address this gap and provide evidence for clinical decision-making and future research directions.

## Materials and methods

### Genome-wide association study datasets

We searched for published genome-wide association studies (GWASs) evaluating individuals of European ancestry on the GWAS Catalog and PubMed (the last search was performed in September 2023). Summary statistics of Ca and P were obtained from the Neale Laboratory with a sample size of 315,153 UK Biobank participants (16). The median, decile 1, and decile 9 values for Ca were 2.376 mmol/L, 2.268 mmol/L, and 2.497 mmol/L, respectively, whereas for P, the corresponding values were 1.165 mmol/L, 0.954 mmol/L, and 1.363 mmol/L. Summary statistics of 25(OH)D traits with a sample size of 417,580, were obtained from the UK Biobank, and the median, mean, and interquartile range of 47.9 mmol/L, 49.6 mmol/L, and 33.5–63.2 nmol/L, respectively (17). Summary statistics of PTH from the INTERVAL study with a sample size of less than 5,000 were used, and the range of PTH was not publicly available (18).

Summary statistics for kidney function traits, including CKD, CKD_Rapid3, CKDi25, eGFRcrea, eGFRcys (estimated glomerular filtration rate using serum cystatin C), BUN (blood urea nitrogen), UACR (urinary albumin–creatinine ratio), and microalbuminuria (MA), were obtained from several published GWAS meta-analyses (19–22). CKD was defined as eGFRcrea of<60 mL min^−1^ per 1.73 m^2^. CKD_Rapid3 was characterized by an annual eGFRcrea decline of 3 mL min^−1^ per 1.73 m^2^ or more and CKDi25 represented a decline of 25% or more in eGFRcrea with a final eGFRcrea below 60 mL min^−1^ per 1.73 m^2^, among individuals with an initial eGFRcrea exceeding 60 mL min^−1^ per 1.73 m^2^. MA was defined as UACR > 25 mg/g in women and > 17 mg/g in men. The majority of individual studies included in these meta-analyses were population-based, with less than 10% originating from diabetic populations, representing less than 5% of the total population in each meta-analysis. The GWASs for CKD, CKD_Rapid3, CKDi25, eGFRcrea, eGFRcys, BUN, UACR, and MA, included a subset of participants, representing 0.16%, 5.8%, 7.7%, 0.16%, 0.16%, 0.16%, 0%, and 0% respectively, from the UK Biobank. These subsets of participants overlapped with those included in GWAS analyses for other exposure factors. Detailed information regarding these studies can be found in **Supplementary Table S1** or by referring to the original studies.

As all these GWASs were approved by their respective local research ethics committees and institutional review boards, no additional ethical approval was necessary for this study.

### LDSC regression analysis

LDSC regression regressed SNV GWAS χ2 statistics for one phenotype to infer SNV-based heritability (h2) or χ2 statistics cross products for a pair of phenotypes to infer SNV-based coheritability on LDSC scores (23). To ensure sufficient statistical power, only phenotypes with a heritability z-score > 4, indicating a substantial proportion of phenotype variance explained by heredity, were included in subsequent analyses. The presence of an intercept close to 1, indicating polygenicity rather than population stratification or cryptic relatedness among samples, accounted for the majority of the observed increase in mean χ2 statistics. The LDSC v1.0.1 software (https://github.com/bulik/ldsc) was employed for this purpose.

### Pleiotropic analyses

#### Pleiotropy at the gene level

Firstly, we assigned SNPs from GWAS summary statistics to genes via an annotation window of ±500 kb flanking the gene boundaries derived from the matched Ensembl build GRCh37 and 1000 G data (24) and then calculated *P-*values for each gene using the multimarker analysis of genomic annotation (MAGMA) v1.09 software (https://ctg.cncr.nl/software/magma) (25). A gene was considered related to a trait if the *P-*value was less than 0.05/the number of all annotated genes or five/the number of all annotated genes (if the number of significant genes was less than five). The next step was to calculate the proportion of genes that were simultaneously associated with both exposure factors and outcomes compared with the total number of genes associated with exposure factors. Additionally, we identified that the SNPs within these genes exhibited horizontal pleiotropy.

#### Pleiotropy at the level of LD-independent loci

The analysis employed the GWAS-PW v2.0.1 software (https://github.com/joepickrell/gwas-pw) (26), and posterior probabilities (PPA) were calculated for 1,703 predefined LD-independent loci (27) to support four scenarios for each locus: [i] the association only to exposure factor (PPA1), [ii] the association only to outcome (PPA2), [iii] shared association to both exposure and outcome via the same SNP (PPA3), and [iv] shared association to exposure and outcome but via two distinct SNPs (PPA4). A PPA3 ≥ 0.9 was used to define a pleiotropic-associated locus. The proportion of the independent loci that influenced the exposure factor also influenced the outcome (ppa3/[ppa1+ppa3+ppa4]) was calculated to determine the possibility of pleiotropy between each exposure and outcome. SNPs meeting the threshold of the third scenario were considered potential horizontal pleiotropic SNPs.

### Two-sample Mendelian randomization analyses

#### Assumptions

**Figure 1:**
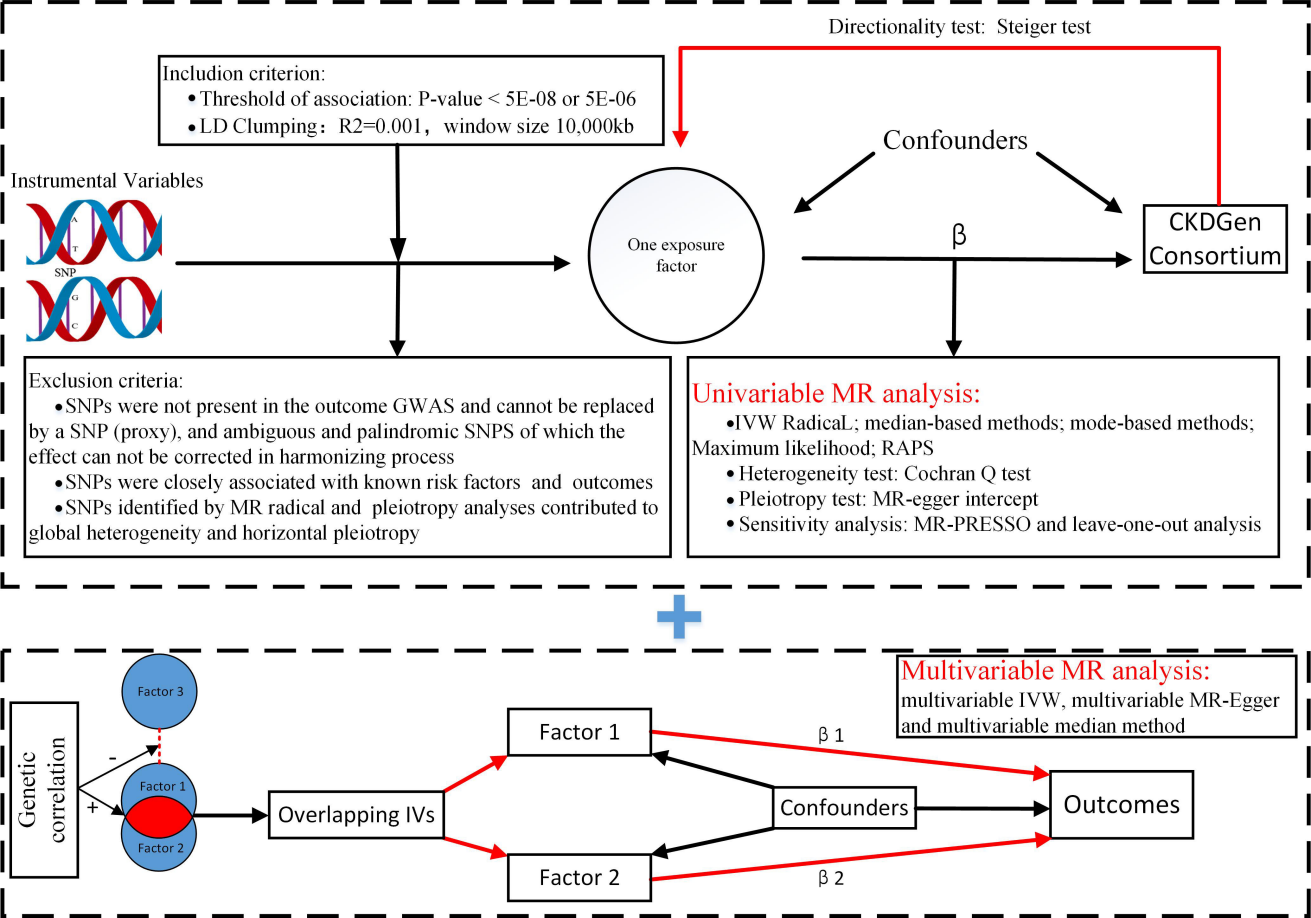
Flowchart of Mendelian randomization analysis. MR, Mendelian randomization; IVW, inverse variance weighted; RAPS, Robust Adjusted Profile Score; MR-PRESSO, Mendelian randomization pleiotropy residual sum and outlier; Lasso regression, least absolute shrinkage and selection operator regression.

The MR analysis (**Figure 1**) was based on the following assumptions: the genetic variants used as instrumental variables (IVs) were associated with the exposure factor; the genetic variants were not associated with any confounders; and the genetic variants were associated with kidney traits through the exposure factor only, namely, non-horizontal pleiotropic variants. Although a direct test for the latter two assumptions was impossible, the analysis methods introduced below could relax these assumptions, serving as a sensitivity analysis.

### Univariable Mendelian randomization analysis

The first step in univariable MR analysis was to construct effective IVs. As some exposure factors had a limited number of candidate IVs (*P-*value < 5E−08) or more than 65% of those (*P-*value < 5E−08) were not available in the GWAS results of the specific outcome, a relatively relaxed significance threshold was set at 5E−06 to improve statistical power. Hence, the first assumption of MR was satisfied. Subsequently, these IVs were grouped based on the 1000 Genomes Project LD structure and independent SNPs (R2 < 0.001 with any other SNP within 10,000 kb) with the most significant *P-*value. Moreover, to satisfy the latter two assumptions as much as possible, IVs associated with known confounders, such as hypertension, diabetes mellitus, lipid traits, body mass index, and gout, available at http://www.phenoscanner.medschl.cam.ac.uk/, were excluded. Following the removal of palindromic SNVs with intermediate allele frequencies, the IVs related to kidney traits (*P-*value [kidney traits] < *P-*value [exposure factors]), and weak IVs with an F value (beta2/se2) of <10, the initially selected IVs were constructed.

The next step was to perform an MR analysis with the initially selected IVs. In the absence of heterogeneity and pleiotropy, the IVW (inverse variance weighted) method combined information on all uncorrelated IVs into a single causal estimate with larger weights given to more precise IVs based on their inverse variances. In particular, the IVW radial as the main approach improved the performance of the IVW estimate and Cochran’s Q statistics by using modified second-order weights (28). Heterogeneity was checked with the Cochran Q test and pleiotropy was tested with MR-Egger. The median-based and median-based approaches were conducted as a supplement given significant heterogeneity, whereas the Robust Adjusted Profile Score (RAPS) (29), which addressed idiosyncratic pleiotropy by robustifying the adjusted profile score, and MR-Egger were conducted as a supplement given significant pleiotropy. Causal directionality was evaluated with the Steiger test (30). The symmetrical funnel plot was generated to identify bias. Additionally, sensitivity analyses were conducted, such as the leave-one-out analysis with the IVW method to check whether the overall estimate was driven by a single SNP. In instances of substantial heterogeneity, the Mendelian Randomization Pleiotropy Residual Sum and Outlier (MR-PRESSO) method was employed for sensitivity analysis. This approach assessed heterogeneity by iteratively excluding each variant to identify horizontal pleiotropic outliers using the Residual Sum of Squares (RSS) (global test). Moreover, the method addressed horizontal pleiotropy by eliminating outliers and examined for significant differences in causal estimates before and after outlier removal (distortion test). When the global test results were significant, the corrected results of MR-PRESSO were considered more reliable than the raw results of that (31).

To further mitigate the risk of false positives, global heterogeneity, and reverse causality, pleiotropic SNPs identified from the pleiotropic analyses, outliers detected by IVW radial (28), and IVs potentially associated with kidney traits (with a *P-*value [kidney traits] < 0.05 if the IV threshold was 5E−08) were excluded to establish the final set of IVs. Subsequently, a new MR analysis was conducted using these final IVs, and the results were compared with those obtained using the initially selected IVs.

### Multivariable Mendelian randomization analysis

The multivariable MR analysis included sets of exposure factors that exhibited genetic correlations among them. IVs were constructed by using multiple sets of overlapping SNPs from the GWASs for relevant exposure factors, which met the univariable MR inclusion criteria described previously. In the absence of heterogeneity and pleiotropy, the multivariable IVW method was the primary choice. If pleiotropy was detected, the multivariable Egger method was used, whereas the multivariable median method was recommended for addressing heterogeneity. Similar to univariable MR, we conducted multivariable MR analyses before and after removing pleiotropic SNPs.

The MR analyses mentioned were conducted using R software v4.1.0 and the following R packages: “TwoSampleMR” v0.5.5, “RadialMR” v2.2.1, and “MendelianRandomization” v0.4.4. The significance threshold for MR analyses mentioned was set at *P-*value = 0.05/12 = 4.2E−03 with Bonferroni correction applied due to the multiple testing (3 exposures × 4 outcomes = 12 tests).

## Results

### LDSC regression

Phenotypes with z-scores < 4, including PTH, CKD_Rapid3, CKDi25, eGFRcys, and MA, were excluded from subsequent analyses (**Table 1**). Genetic correlations were observed between serum 25(OH)D and eGFRcrea (rg −0.094; *P-*value 1.4E−05), as well as BUN (rg 0.127; *P-*value 1.7E−06). Serum Ca showed a significant genetic correlation with UACR (rg 0.202, *P-*value 5.0E−04). However, there was no statistically significant genetic correlation between serum P and CKD (*P-*value 0.03). Among the exposure factors, only serum 25(OH)D was correlated with serum P (rg 0.076; *P-*value 0.002) (**Table 2**).

**Table 1:** LDSC regression estimates of all exposure factors and kidney traits.

Phenotype	Study design	Sample size	h^2^ estimate (SE)	Z-score
Calcium	Population-based	315,153	0.115 (0.010)	11.5
Phosphate	Population-based	315,153	0.108 (0.01)	10.8
25-hydroxyvitamin D	Population-based	417,580	0.096 (0.014)	6.9
Parathyroid hormone	Population-based	3,310	0.137 (0.122)	1.1*
CKD	Meta-analysis	480,698	0.010 (0.001)	10.0
CKD_Rapid3	Meta-analysis	141,964	0.009 (0.003)	3.0*
CKDi25	Meta-analysis	195,145	0.010 (0.0022)	4.5*
eGFRcrea	Meta-analysis	567,460	0.071 (0.004)	17.8
eGFRcys	Meta-analysis	24,061	0.134 (0.055)	2.4*
BUN	Meta-analysis	243,029	0.064 (0.005)	12.8
UACR	Meta-analysis	54,450	0.046 (0.008)	5.8
MA	Meta-analysis	54,116	0.014 (0.007)	2.0*

LDSC, linkage disequilibrium score; h^2^, heritability; CKD, chronic kidney disease; eGFRcrea, estimated glomerular filtration rate using serum creatinine; eGFRcys, estimated glomerular filtration rate using serum cystatin C; BUN, blood urea nitrogen; UACR, urinary albumin-to-creatinine ratio; MA, microalbuminuria.

aλGC refers to the genomic inflation factor. *Z-score < 4.

**Table 2:** Genetic correlation estimates from LDSC regression.

Exposure	Outcome	rg (se)	rg intercept (se)	*P-*value
Calcium	Phosphate	−0.080 (0.049)	0.186 (0.013)	0.10
	25(OH)D	0.032 (0.024)	0.058 (0.010)	0.18
	CKD	0.010 (0.052)	0.016 (0.007)	0.85
	eGFRcrea	0.002 (0.029)	−0.013 (0.009)	0.95
	BUN	−0.034 (0.036)	0.023 (0.008)	0.34
	UACR	0.202 (0.058)	−0.002 (0.006)	5.0E−04*
25(OH)D	Calcium	0.032 (0.024)	0.058 (0.010)	0.18
	Phosphate	0.076 (0.025)	0.039 (0.008)	0.002*
	CKD	0.071 (0.042)	0.009 (0.007)	0.10
	eGFRcrea	−0.094 (0.022)	−0.019 (0.009)	1.4E−05*
	BUN	0.127 (0.027)	0.009 (0.007)	1.7E−06*
	UACR	0.006 (0.052)	−0.004 (0.007)	0.91
Phosphate	Calcium	−0.080 (0.049)	0.186 (0.013)	0.10
	25(OH)D	0.076 (0.025)	0.039 (0.008)	0.002
	CKD	−0.118 (0.055)	0.009 (0.007)	0.03
	eGFRcrea	0.057 (0.035)	−0.006 (0.009)	0.10
	BUN	0.041 (0.037)	0.003 (0.008)	0.27
	UACR	−0.037 (0.060)	0.002 (0.006)	0.54

LDSC, linkage disequilibrium score; 25-hydroxyvitamin D, 25(OH)D; CKD, chronic kidney disease; eGFRcrea, estimated glomerular filtration rate using serum creatinine; BUN, blood urea nitrogen; UACR, urinary albumin-to-creatinine ratio; rg, reflects the strength of the genetic relationship between the traits.

*P-value < 4.2E−03.

### Pleiotropic analyses

**Figure 2:**
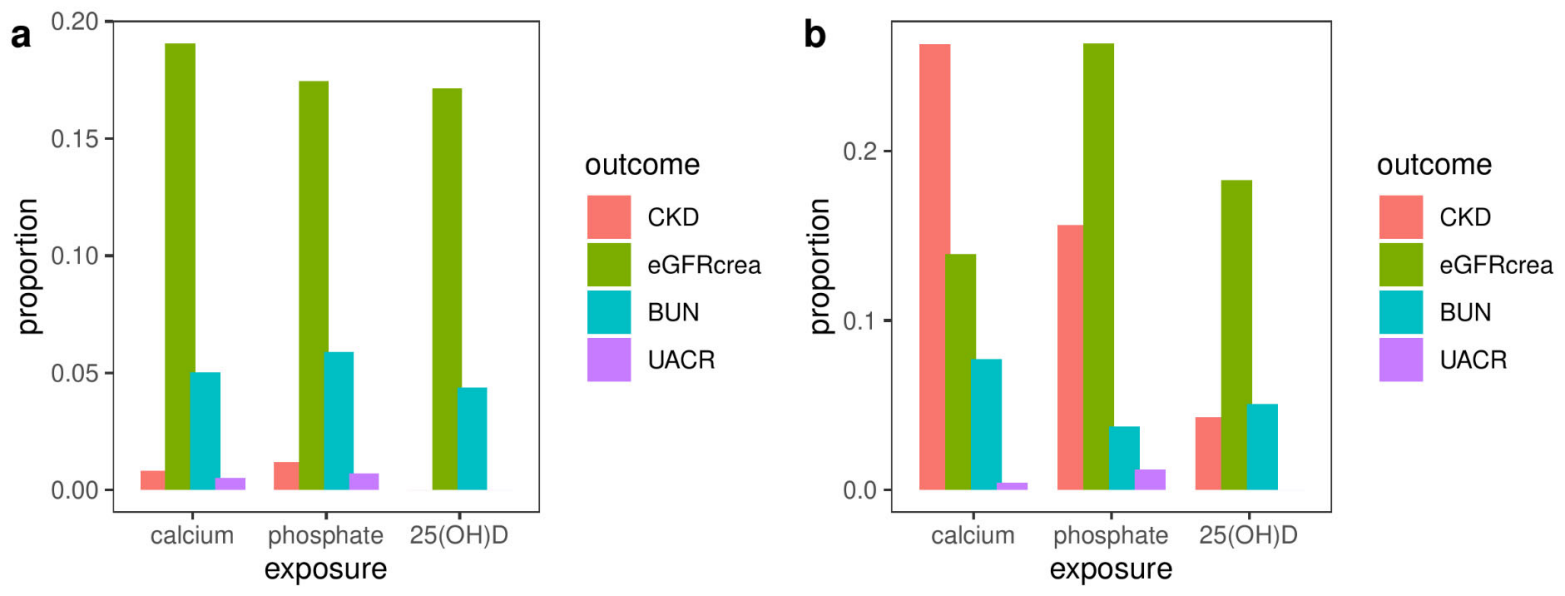
The proportion of horizontally pleiotropic genes **(A)** or loci **(B)** for each exposure among all the genes or loci associated with that particular exposure. CKD, chronic kidney disease; eGFRcrea, estimated glomerular filtration rate using serum creatinine; BUN, blood urea nitrogen; UACR, urinary albumin-to-creatinine ratio.

The characteristics of the annotated genes and related LD-independent loci are described in **Supplementary Tables S2 and S3**. **Figure 2** illustrates the proportion of horizontally pleiotropic genes for each exposure among all the genes associated with that particular exposure, as well as the percentage of horizontally pleiotropic LD-independent loci for each exposure among all the loci associated with that exposure. The potential pleiotropic SNPs located within the pleiotropic genes or loci are presented in **Supplementary Table S4**.

### Two-sample Mendelian randomization analysis

Due to the unavailability of more than 65% of candidate IVs with a *P-*value less than 5E−08 in the GWAS results of UACR, a relatively relaxed significance threshold of 5E−06 was adopted when investigating UACR as the outcome.

### Univariable Mendelian randomization analysis

The characteristics of the initially selected effective IVs after removing confounding IVs (**Supplementary Table S4**), and the final set of effective IVs chosen after additional elimination of pleiotropic IVs (**Supplementary Table S5**), outliers (**Supplementary Table S6**), and IVs associated with the outcome, were detailed in **Supplementary Tables S5–S30**. Over 85% of the variants overlapped between outliers and IVs associated with the outcome.

There was no evidence of a violation of the causal directionality assumption in any of the univariable MR analyses (**Supplementary Tables S31, S32**). Initially selected IVs exhibited widespread heterogeneity but no evidence of horizontal pleiotropy. Upon using the finally selected IVs, the heterogeneity disappeared. However, horizontal pleiotropy was only evident for the relationship between 25(OH)D and BUN (*P-*value 0.04) (**Supplementary Tables S33–S36**). Although the global test of MR-PRESSO identified horizontal pleiotropic outliers only with initially selected IVs, except for the relationships involving Ca or 25(OH)D and UACR, the distortion test did not reveal significant differences in causal estimates before and after the removal of outliers (**Supplementary Tables S37, S38**). All symmetrical funnel plots indicated no disproportionate effects from selected IVs (**Supplementary Figures S1–S4**).

None of the MR analysis methods found any exposure factors associated with higher odds of CKD after the Bonferroni correction (*P-*value>4.2E-03) (**Supplementary Tables S39, 40** and **Supplementary Figures S5, S6**).

**Figure 3:**
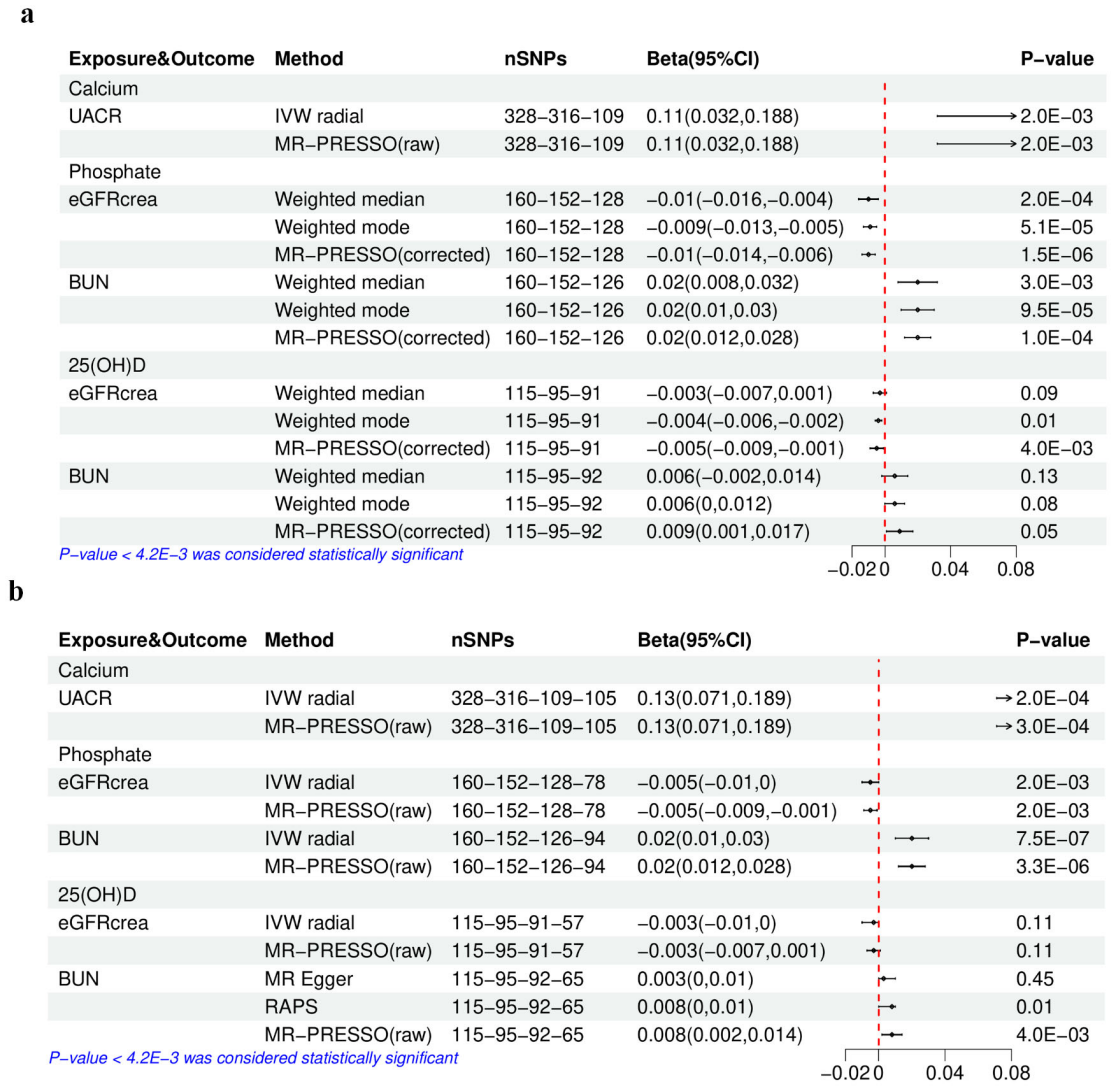
The results of univariable Mendelian randomization analysis of calcium, phosphate, or 25(OH)D–eGFRcrea, BUN, or UACR. **(A)** Using the initially selected IVs; **(B)** using the finally selected IVs. 25(OH)D, 25-hydroxyvitamin D; eGFRcrea, estimated glomerular filtration rate using serum creatinine; BUN, blood urea nitrogen; UACR, urinary albumin-to-creatinine ratio; IVW, inverse variance weighted; RAPS, Robust Adjusted Profile Score; MR-PRESSO, Mendelian randomization pleiotropy residual sum and outlier. The four numbers in the nSNP column represent the remaining IVs after each IV selection step: step 1—threshold of association and LD clumping, step 2—harmony and excluding palindromic SNPs, step 3—deleting IVs with outcome correlation *P-*value less than exposure correlation *P-*value, step 4—removing pleiotropic SNPs, outliers detected by IVW radical, and IVs potentially associated with kidney traits (with a *P-*value [kidney traits] < 0.05 if the IV threshold was 5E−08).

In the analysis conducted with the initially selected IVs (**Supplementary Table S41**), the primary method, weighted median, supported both the associations between P and eGFRcrea (beta −0.01; *P-*value 2.0E−04), as well as between Ca and eGFRcrea (beta 0.009; *P-*value 8.0E−04). This finding was further confirmed by the main sensitivity analysis, MR-PRESSO (**Figure 3**) (**Supplementary Table S37**), rather than the leave-one-out analysis (**Supplementary Figure S7**). In the analysis utilizing the finally selected IVs (**Supplementary Table S42**), the IVW radial method consistently affirmed the association between P and eGFRcrea (beta −0.005; *P-*value 2.0E−03), supported by sensitivity analyses with MR-PRESSO (**Figure 3**) and leave-one-out analysis (**Supplementary Figure S8**).

Causal estimates for BUN (**Supplementary Tables S43, 44**) indicated that with the initially selected IVs, only higher serum P was linked to a higher BUN, as evidenced by the weighted median (beta 0.02; *P-*value 3.0E−03), as well as sensitivity analyses using the MR-PRESSO (**Figure 3**) and the leave-one-out analysis (**Supplementary Figure S9**). Furthermore, in the analysis with the final IV selection, the primary analysis method, IVW radial approach, also validated the causal relationship between P and BUN (beta 0.02; *P-*value 7.5E−07), supported by sensitivity analyses using MR-PRESSO (**Figure 3**) and leave-one-out analysis (**Supplementary Figure S10**).

Regarding UACR (**Supplementary Tables S45, S46**), in the analysis using either the initially selected or the finally selected IVs, it was found that only genetically predicted higher Ca was related to the increase in UACR, as confirmed by the IVW radial method (beta 0.11, *P-*value 2.0E−03; beta 0.13, *P-*value 2.0E−04) and sensitivity analyses using the MR-PRESSO (**Figure 3**) and the leave-one-out analysis (**Supplementary Figures S11, S12**).

### Multivariable Mendelian randomization analysis

**Figure 4:**
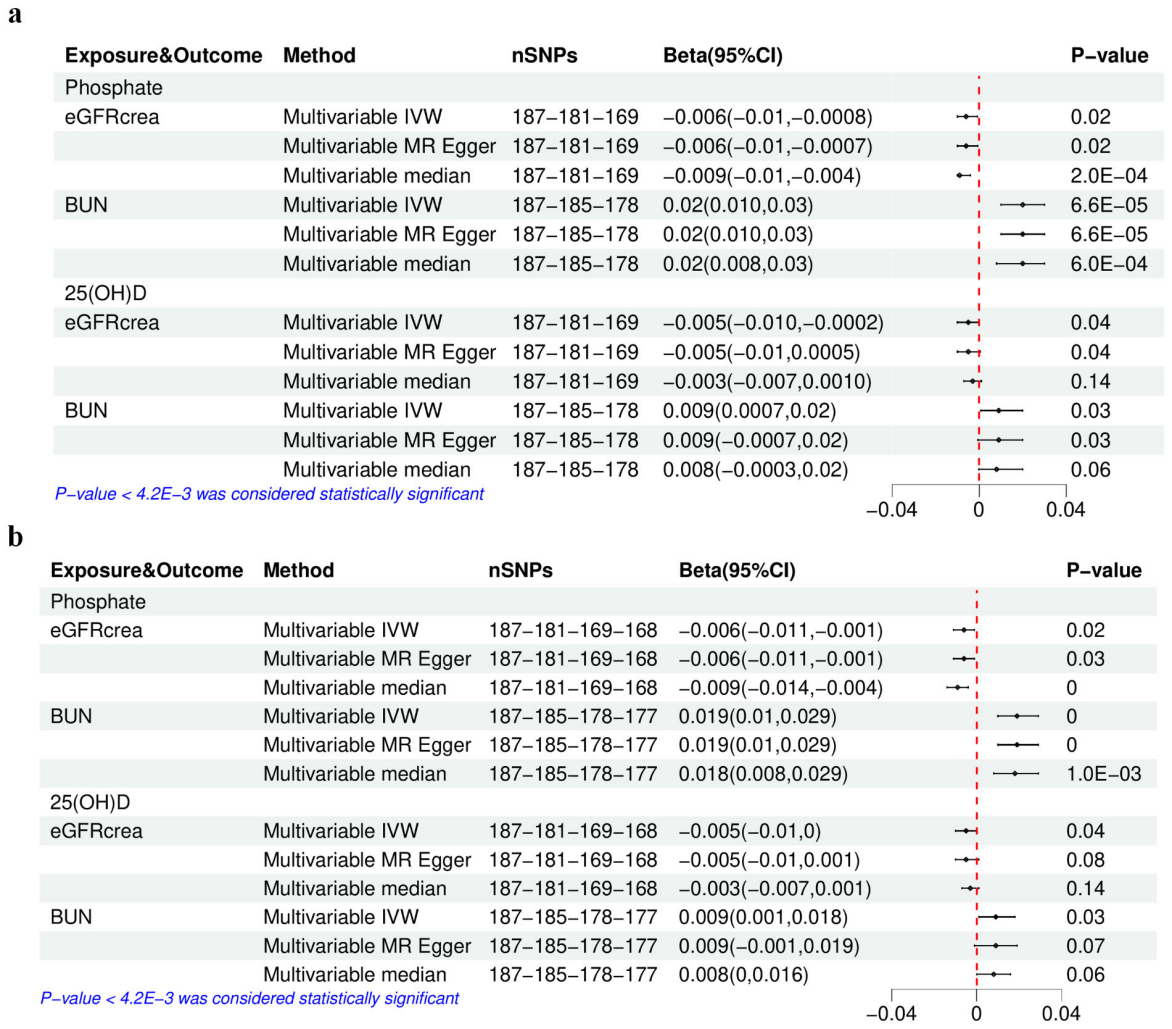
The results of multivariable Mendelian randomization analysis of serum phosphate and 25(OH)D–eGFRcrea or BUN. **(A)** Using the initially selected IVs; **(B)** using the finally selected IVs. IVW, inverse variance weighted; 25(OH)D, 25-hydroxyvitamin D; eGFRcrea, estimated glomerular filtration rate using serum creatinine; BUN, blood urea nitrogen. The four numbers in the nSNP column represent the remaining IVs after each IV selection step: step 1—threshold of association and LD clumping, step 2—harmony and excluding palindromic SNPs, step 3—deleting IVs with outcome correlation *P-*value less than exposure correlation *P-*value, step 4—removing pleiotropic SNPs.

The concurrent utilization of serum P and 25(OH)D as exposure variables in a multivariable MR analysis was driven by the identified genetic correlation between the two. The characteristics of the selected SNPs after excluding confounding IVs (**Supplementary Table S47**) and those finally selected after further removing pleiotropic IVs (**Supplementary Table S48**) are detailed in **Supplementary Tables S49–S54**. Both the initial and final IV sets showed extensive heterogeneity but no evidence of horizontal pleiotropy (**Supplementary Tables S55, S56**). The multivariable median method considered as the main method only showed that the associations between serum P and eGFRcrea or BUN were still significant (*P-*value < 4.2E-03) (**Figure 4**), regardless of the exclusion of pleiotropic IVs, consistent with the results in univariable MR analysis.

## Discussion

Controversy exists regarding whether serum Ca, P, 25(OH)D, and PTH contribute to the deterioration of renal function, and clarifying these associations is essential for both nephrologists and the general population.

Although genetic correlations between 25(OH)D and eGFRcrea or BUN were significant, both univariable and multivariable MR analyses in our study indicated that these correlations did not represent causal relationships. This finding was consistent with a previous MR study (15) that only conducted univariable MR analysis. The reason behind this lack of causality can be attributed to the fact that the serum 25(OH)D levels were associated with various health conditions beyond serum Ca and P levels, including diseases closely related to renal function such as glucose metabolism disorders, weight gain, infectious diseases, immune function, and cancer (32). The extensive phenotypic effects of genetic variants in the GWAS data for 25(OH)D that did not meet the threshold for MR resulted in the genetic correlation between 25(OH)D and kidney function. Similarly, clinical studies influenced or mediated by these conditions may not yield reliable results.

Interestingly, our findings revealed a causal and detrimental relationship between serum Ca and UACR, but no such relationship was observed with BUN. Despite the absence of genetic correlations between serum Ca and eGFRcrea, the use of initially selected IVs in MR analysis, rather than finally selected IVs, supported this causal effect. Notably, a high proportion (92.5%) of outliers identified by the IVW radical among the initially selected IVs were potentially associated with the outcome (*P-*value < 0.05), suggesting potential bias if these IVs were included in the MR analysis. Moreover, excluding these outcome-related IVs led to non-significant results (nSNPs 112, beta 0.002, *P-*value 0.20) aligning with the analysis with finally selected IVs. Consequently, the causal effect of Ca on eGFRcrea cannot be conclusively established.

**Figure 5:**
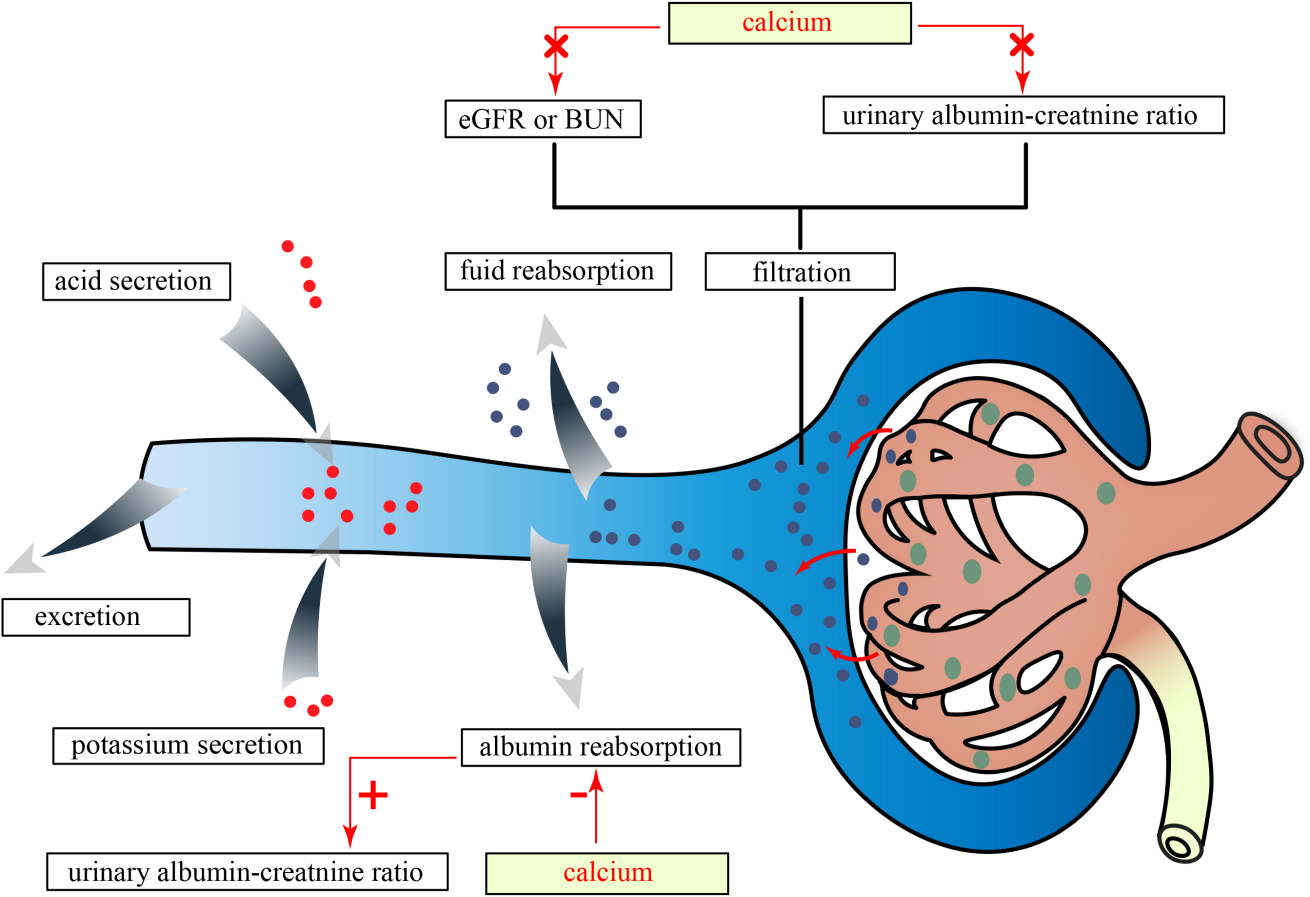
The effects of serum Ca on kidney traits in this study. eGFRcrea, estimated glomerular filtration rate using serum creatinine; BUN, blood urea nitrogen; Ca, calcium; P, phosphate.

Considering the evidence that the normal kidney filtered nephrotic levels of albumin retrieved by proximal tubule cells (33), our findings suggested that serum Ca might only damage the albumin reabsorption function of PTEC instead of the surface area of the glomerular capillary wall, and its permeability to small solutes and water mainly determined eGFRcrea and BUN (**Figure 5**). *In vitro* studies have shown that elevated extracellular Ca for 18 h–20 h could induce damage in human PTEC (34). Another study demonstrated that high Ca levels for 24 h, through the activation of reactive oxygen species derived from nicotinamide adenine dinucleotide phosphate oxidase subunit 4 via protein kinase C, caused oxidative stress damage and apoptosis in PTECs (35). However, short-term activation of PTEC-specific CaSR (calcium-sensing receptor) by perfusion with varying Ca concentrations for 2 to 8 min only enhances fluid reabsorption without causing damage (36). These findings suggested that the duration of high Ca exposure had different effects on PTECs. *In vivo*, a study suggested that long-term inactivation of CaSR may protect renal function. Specifically, treatment with the CaSR antagonist ronacaleret for 3 months can help maintain the expression of klotho and nephrin, preserve renal function, and reduce serum P and proteinuria in rats with 5/6 nephrectomy without affecting blood pressure, PTH, 1,25(OH)D, or serum Ca levels (37). However, another study supporting the renal protective effects of CaSR activation showed that cinacalcet reduced toxin-induced proteinuria and glomerular damage in mice on the 10th and 12th days after a 2-day administration (38). We speculated that this protective effect might be due to decreased renin activity induced by acute CaSR activation with 2-day treatment, which was independent of PTH and could not be induced by chronic CaSR activation over 7 days in juxtaglomerular cells (39). Based on the results of our MR analyses and in consideration of prior research, we speculated that long-term elevation of serum Ca may impair kidney function through the activation of CaSR.

Research conducted on animal models of CKD has provided substantial evidence for a significant association between the intake of dietary P and the progression of CKD (40, 41). Previous studies have found that the mediation of serum P signaling by type III sodium-dependent phosphate transporters occurred independently of their P transport function, possibly involving downstream modulation of ERK signaling, thereby contributing to glomerular damage (42). Our study employing univariable MR analysis and multivariable MR analysis in conjunction with 25(OH)D has consistently supported the causal effect of serum P on eGFRcrea or BUN. Nevertheless, the effect size (beta −0.005) was relatively small, indicating that larger sample sizes and longer follow-up durations may be required to achieve sufficient statistical power in RCT. This could explain why the effects of serum P on kidney function have not been observed in an RCT with 31 participants and a 3-week follow-up period (43).

There were some limitations in the present study: (1) Our findings may not be generalizable to other ancestries due to the GWAS dataset being sourced solely from European populations; (2) due to low heritability, several related traits could not be included in the MR analysis using existing GWAS studies; (3) despite efforts to mitigate population specificity, population stratification, and potential sample overlap, it was not possible to completely eliminate these factors; (4) our findings have not been verified through experimental studies, as this was beyond the scope of our study. Therefore, further research should be designed to address these limitations.

## Conclusions

Based on the genetic analyses conducted in our study, it was crucial to closely monitor serum Ca levels to prevent potential kidney toxicity resulting from elevated serum Ca during the administration of Ca supplements or vitamin D therapy. Additionally, it was important to acknowledge the progressive decline in residual renal function that can occur due to high serum phosphate levels. Finally, there is no evidence to suggest that vitamin D analogs directly improve renal function.

## Data Availability

The original contributions presented in the study are included in the article/**Supplementary Material**. Further inquiries can be directed to the corresponding author.

## Glossary

MR: Mendelian randomization
LDSC: linkage disequilibrium score
MAGMA: multimarker analysis of genomic annotation
GWAS: genome-wide association studies
SNV: single-nucleotide variant
SNP: single-nucleotide polymorphism
h2: heritability
IVs: instrumental variables
IVW: inverse variance weighted
MR-PRESSO: Mendelian randomization pleiotropy residual sum and outlier
RAPS: Robust Adjusted Profile Score
Ca: calcium
P: phosphate
ALP: alkaline phosphatase
25(OH)D: 25-hydroxyvitamin D
Mg: magnesium
PTH: parathyroid hormone
FGF23: fibroblast growth factor 23
EPO: erythropoietin
CKD: chronic kidney disease
eGFRcrea: estimated glomerular filtration rate using serum creatinine
eGFRcys: estimated glomerular filtration rate using serum cystatin C
BUN: blood urea nitrogen
UACR: urinary albumin–creatinine ratio
MA: microalbuminuria
CaSR: calcium-sensing receptor.
