# Supplementary material for: Causal effects of serum calcium, phosphate, and 25-hydroxyvitamin D on kidney function: a genetic correlation, pleiotropic analysis, and Mendelian randomization study
Source: Front Endocrinol (Lausanne). 2024 Sep 30;15:1348854. doi: 10.3389/fendo.2024.1348854 (PMC11471720; doi:10.3389/fendo.2024.1348854)
Supplement: Supplementary file 1 [file DataSheet1.zip › Supplementary Figure.pdf]

Figure S1. Symmetrical funnel plots of the relationship between each exposure factor and CKD.

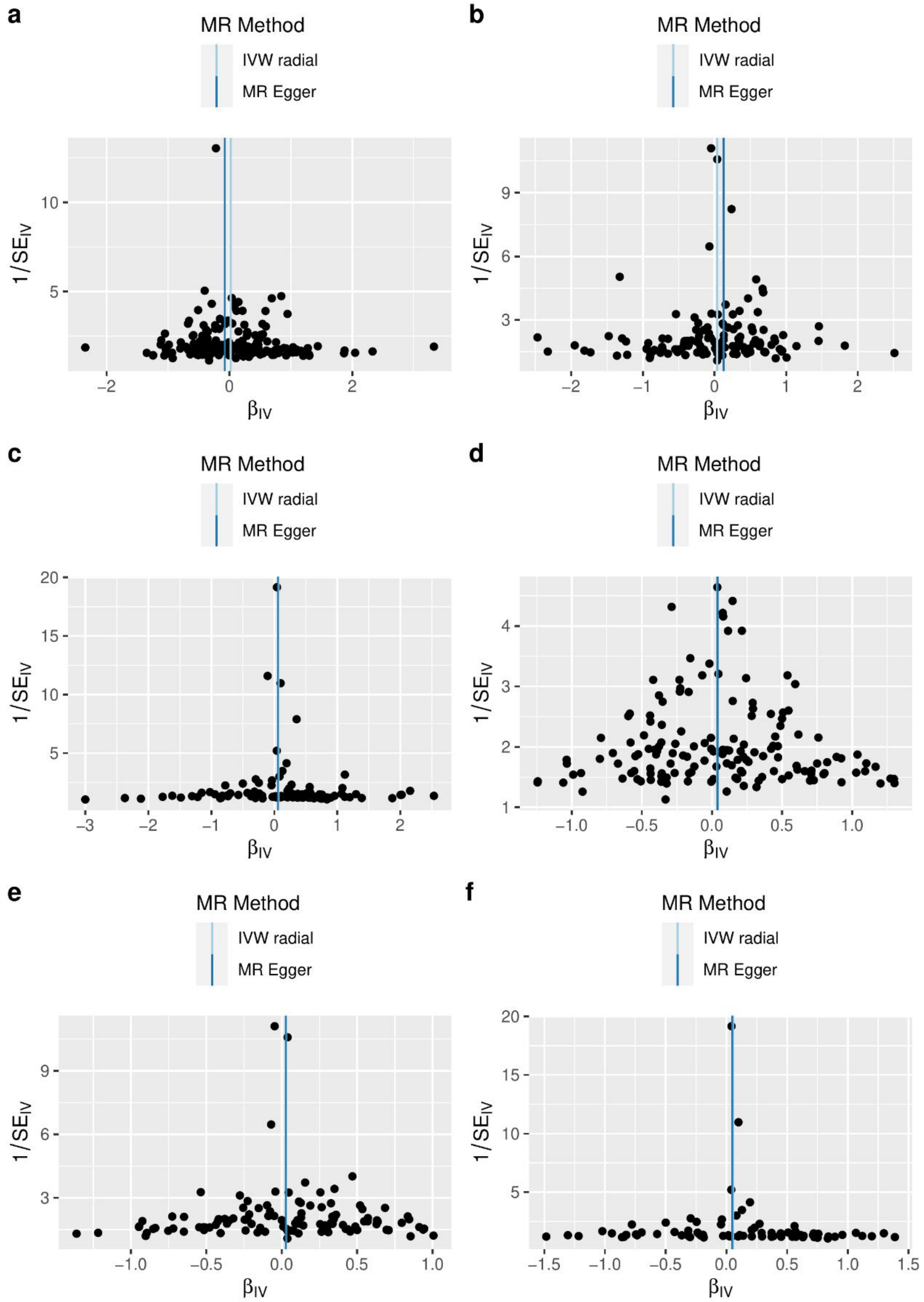

Figures a-c show funnel plots for the calcium (a), phosphate (b), 25- hydroxyvitamin D (c), and CKD relationship using initially selected IVs. Figures d-f display funnel plots for the calcium (d), phosphate (e), 25- hydroxyvitamin D (f), and CKD relationship using finally selected IVs.

CKD, chronic kidney disease; IVs: instrumental variables.

Figure S2. Symmetrical funnel plots of the relationship between each exposure factor and eGFRcrea.

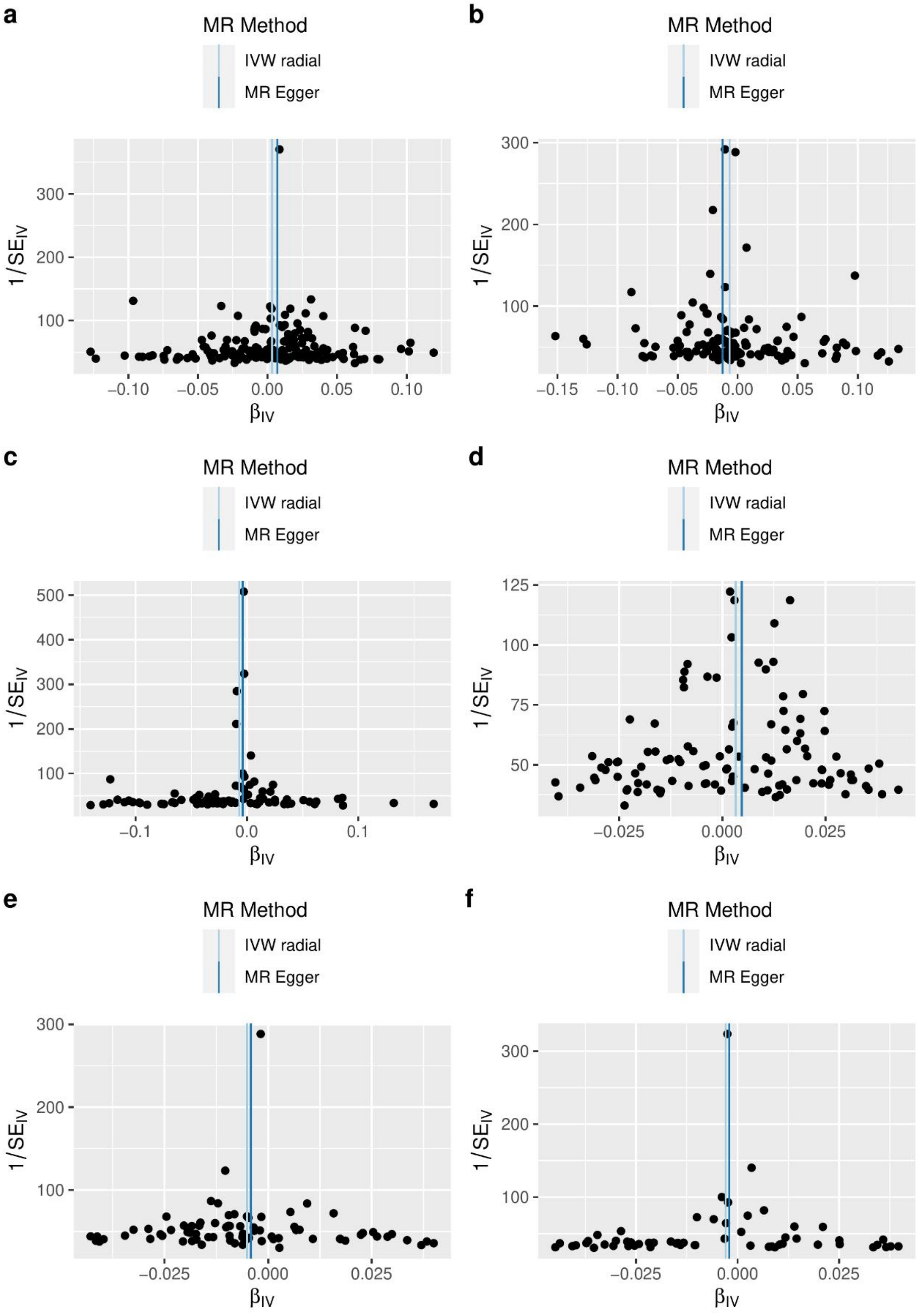

Figures a-c show funnel plots for the calcium (a), phosphate (b), 25- hydroxyvitamin D (c), and eGFRcrea relationship using initially selected IVs. Figures d-f display funnel plots for the calcium (d), phosphate (e), 25- hydroxyvitamin D (f), and eGFRcrea relationship using finally selected IVs.

eGFRcrea, estimated glomerular filtration rate based on serum creatinine; IVs: instrumental variables.

Figure S3. Symmetrical funnel plots of the relationship between each exposure factor and BUN.

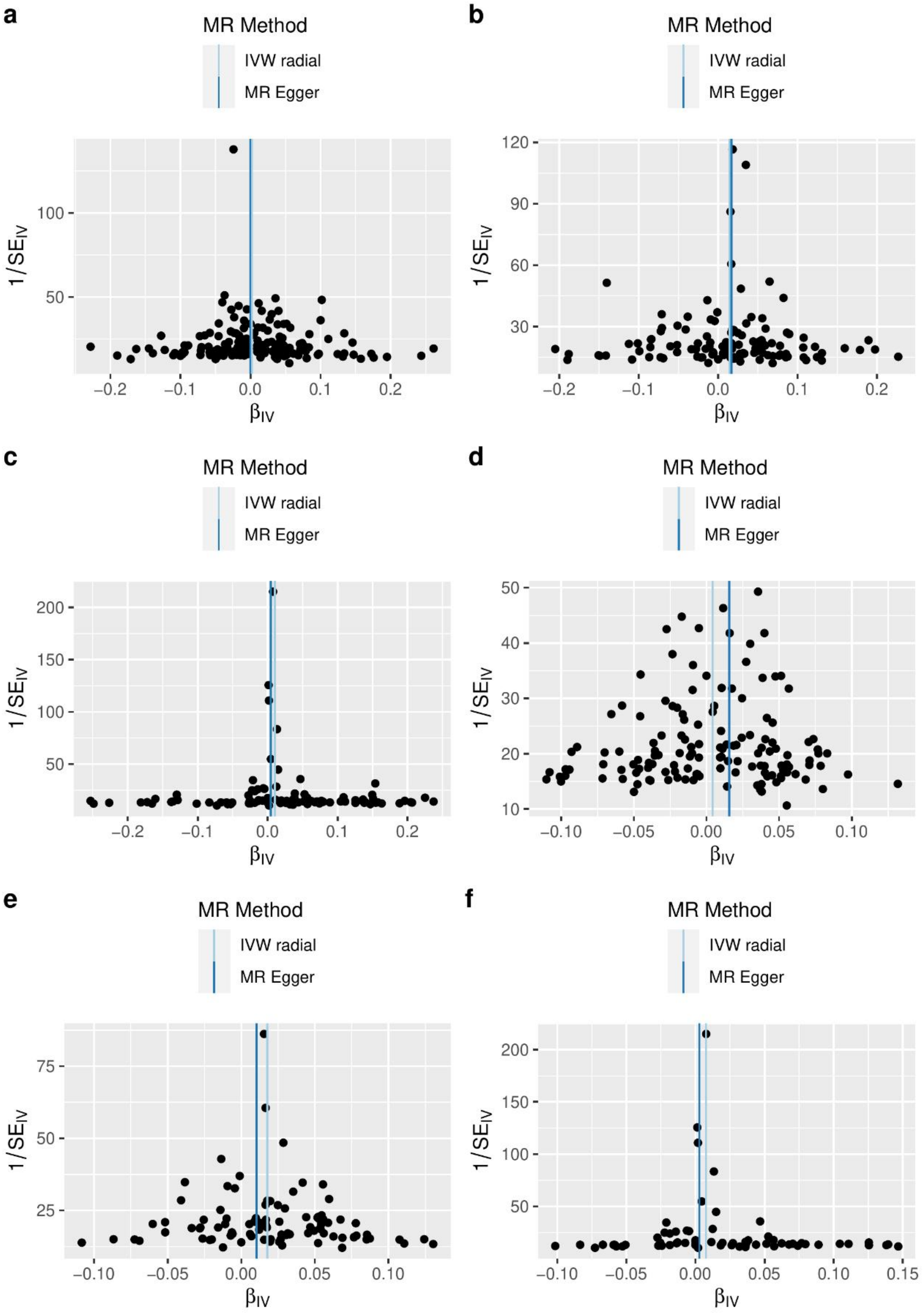

Figures a-c show funnel plots for the calcium (a), phosphate (b), 25- hydroxyvitamin D (c), and BUN relationship using initially selected IVs. Figures d-f display funnel plots for the calcium (d), phosphate (e), 25- hydroxyvitamin D (f), and BUN relationship using finally selected IVs.

BUN, blood urea nitrogen; IVs: instrumental variables.

Figure S4. Symmetrical funnel plots of the relationship between each exposure factor and UACR.

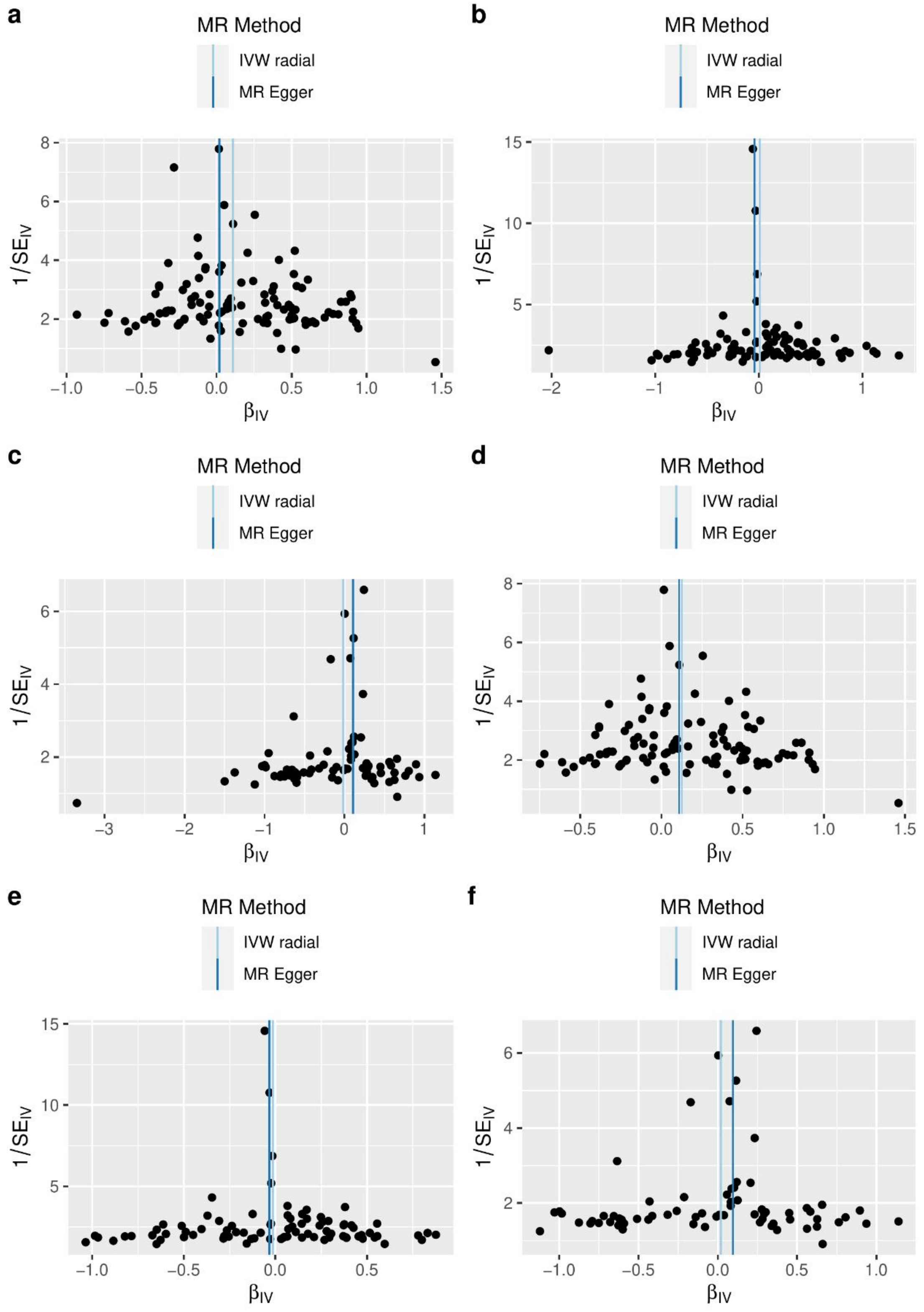

Figures a-c show funnel plots for the calcium (a), phosphate (b), 25- hydroxyvitamin D (c), and BUN relationship using initially selected IVs. Figures d-f display funnel plots for the calcium (d), phosphate (e), 25- hydroxyvitamin D (f), and BUN relationship using finally selected IVs.

UACR, urinary albumin-to-creatinine ratio; IVs: instrumental variables.

Figure S5. Plots of leave-one-out analyses of the relationship between each exposure factor and CKD using initially selected instrumental variables.

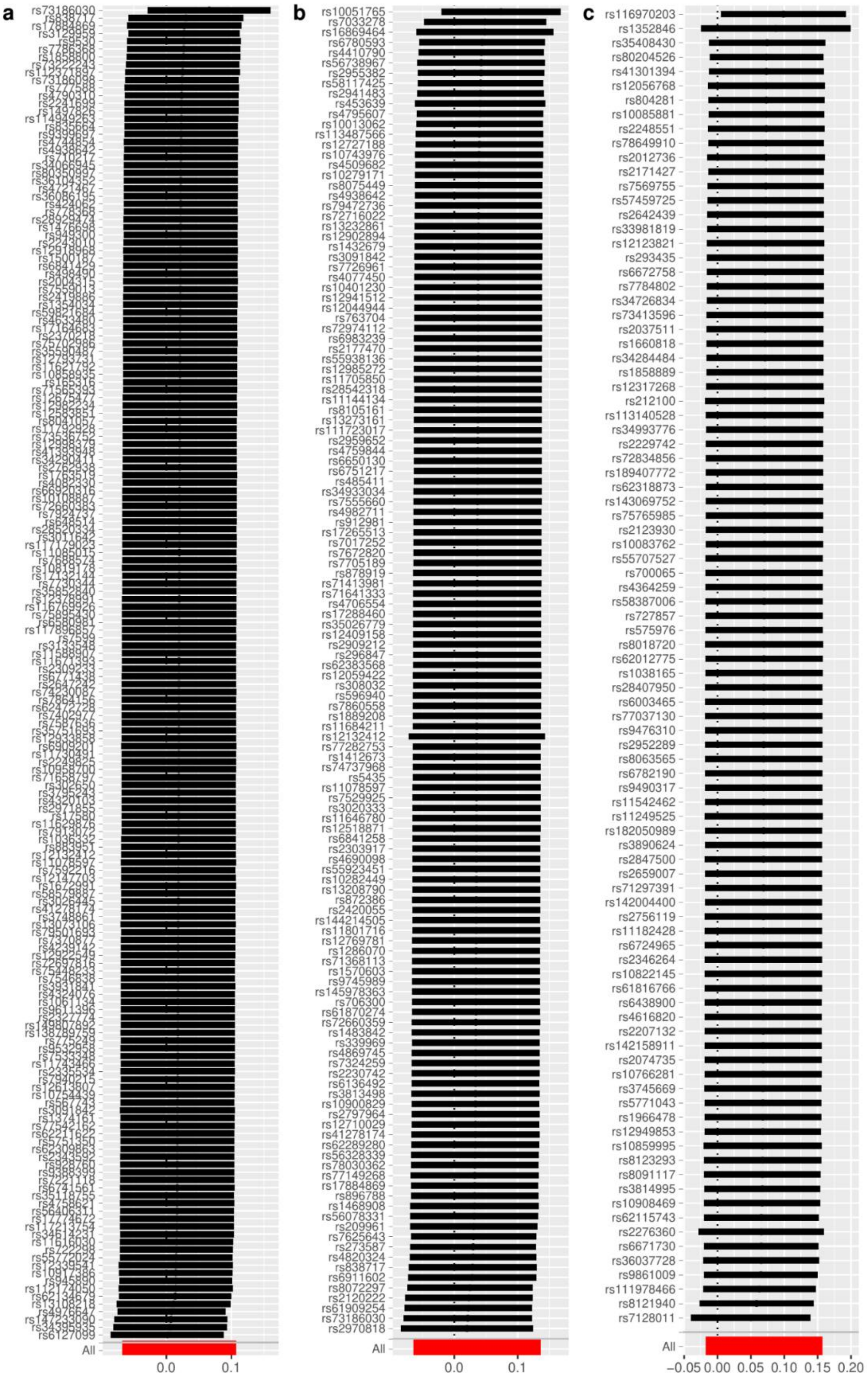

(a) Exposure: calcium; Outcome: CKD; (b) Exposure: phosphate; Outcome: CKD; (c) Exposure: 25-hydroxyvitamin D; Outcome: CKD. CKD, Chronic kidney disease.

Figure S6. Plots of leave-one-out analyses of the relationship between each exposure factor and CKD using finally selected instrumental variables.

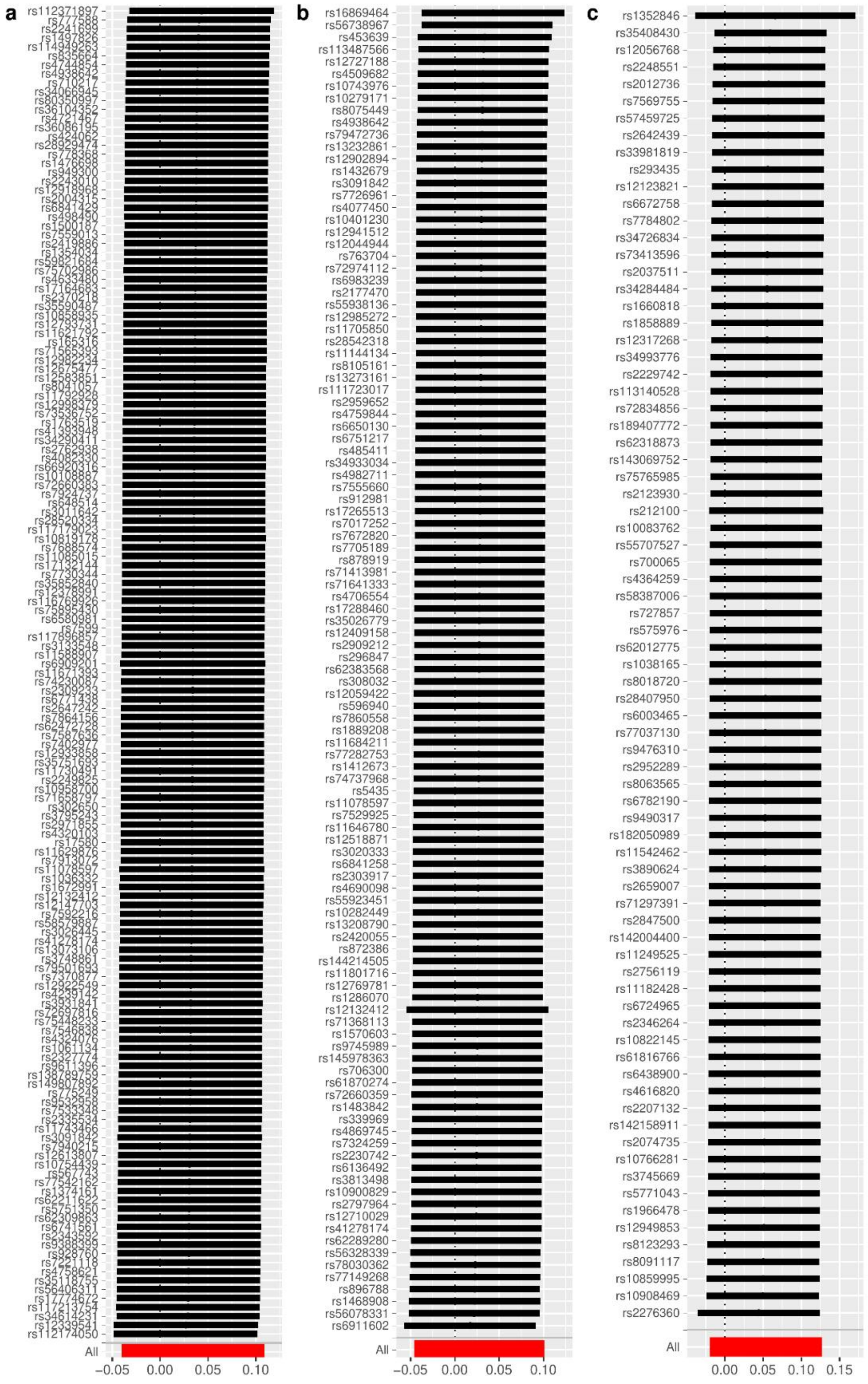

(a) Exposure: calcium; Outcome: CKD; (b) Exposure: phosphate; Outcome: CKD; (c) Exposure: 25-hydroxyvitamin D; Outcome: CKD. CKD, Chronic kidney disease.

Figure S7. Plots of leave-one-out analyses of the relationship between each exposure factor and eGFRcrea using initially selected instrumental variables.

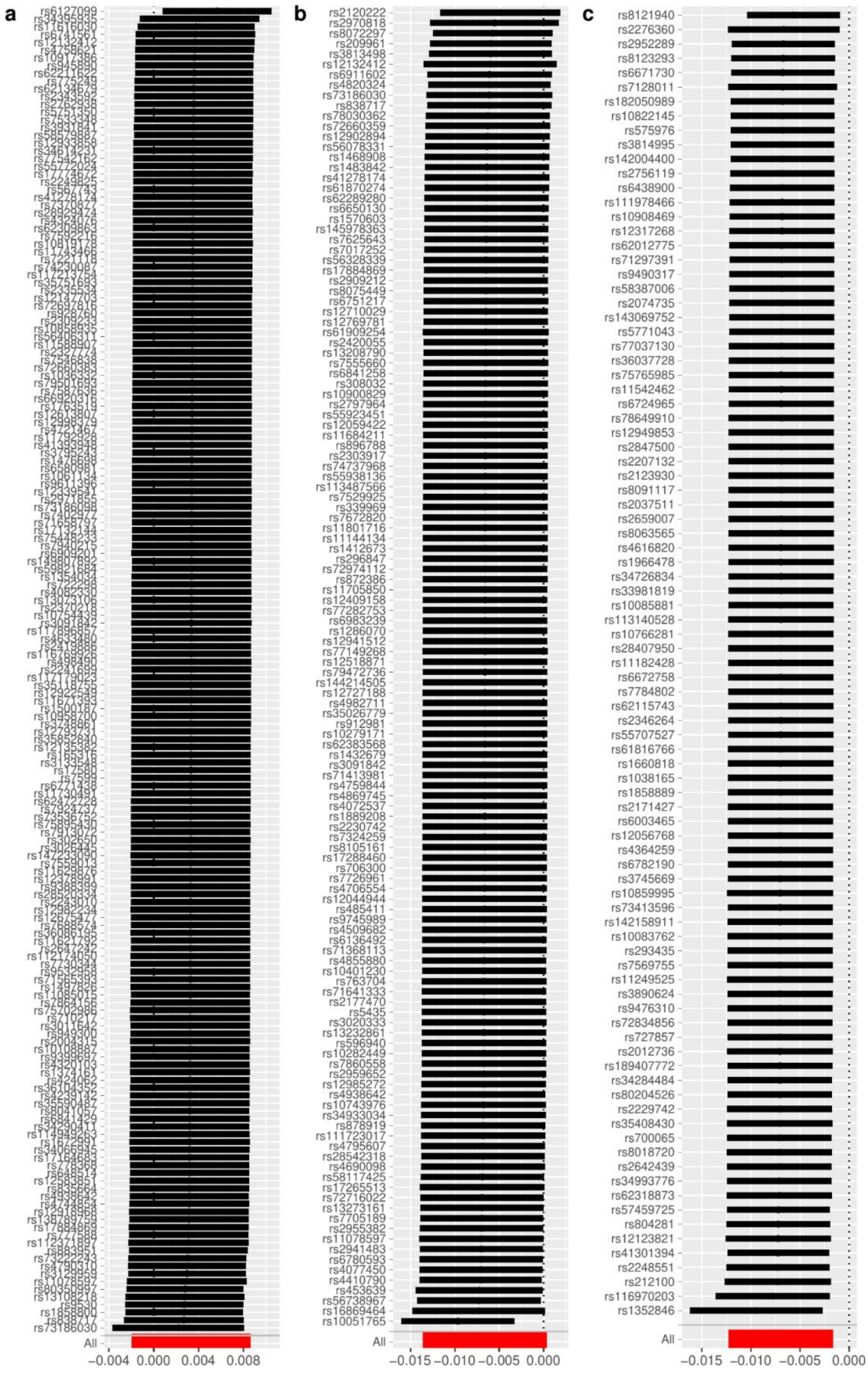

(a) Exposure: calcium; Outcome: eGFRcrea; (b) Exposure: phosphate; Outcome: eGFRcrea; (c) Exposure: 25- hydroxyvitamin D; Outcome: eGFRcrea. eGFRcrea, estimated glomerular filtration rate based on serum creatinine.

Figure S8. Plots of leave-one-out analyses of the relationship between each exposure factor and eGFRcrea using finally selected instrumental variables.

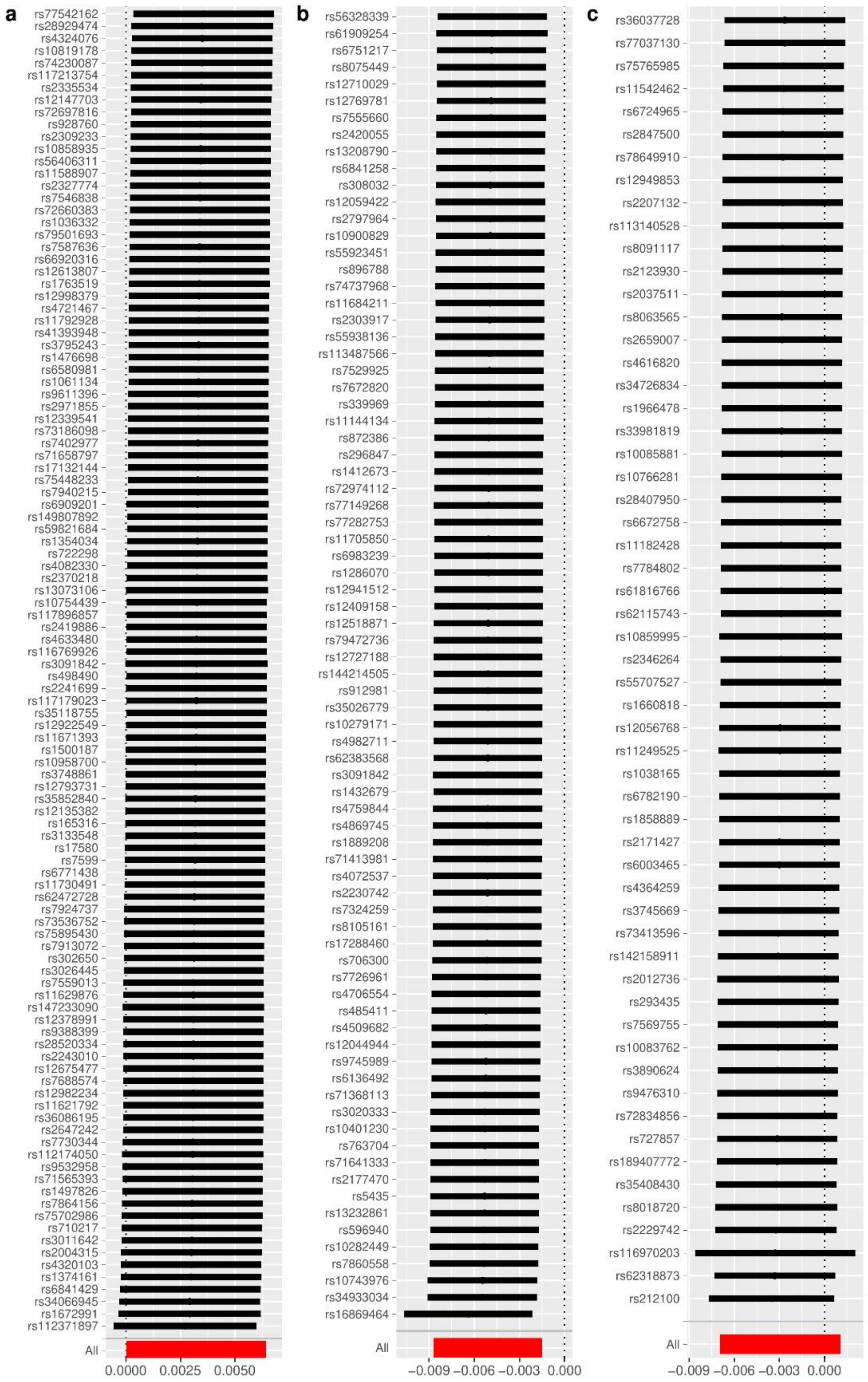

(a) Exposure: calcium; Outcome: eGFRcrea; (b) Exposure: phosphate; Outcome: eGFRcrea; (c) Exposure: 25- hydroxyvitamin D; Outcome: eGFRcrea. eGFRcrea, estimated glomerular filtration rate based on serum creatinine.

Figure S9. Plots of leave-one-out analyses of the relationship between each exposure factor and BUN using initially selected instrumental variables.

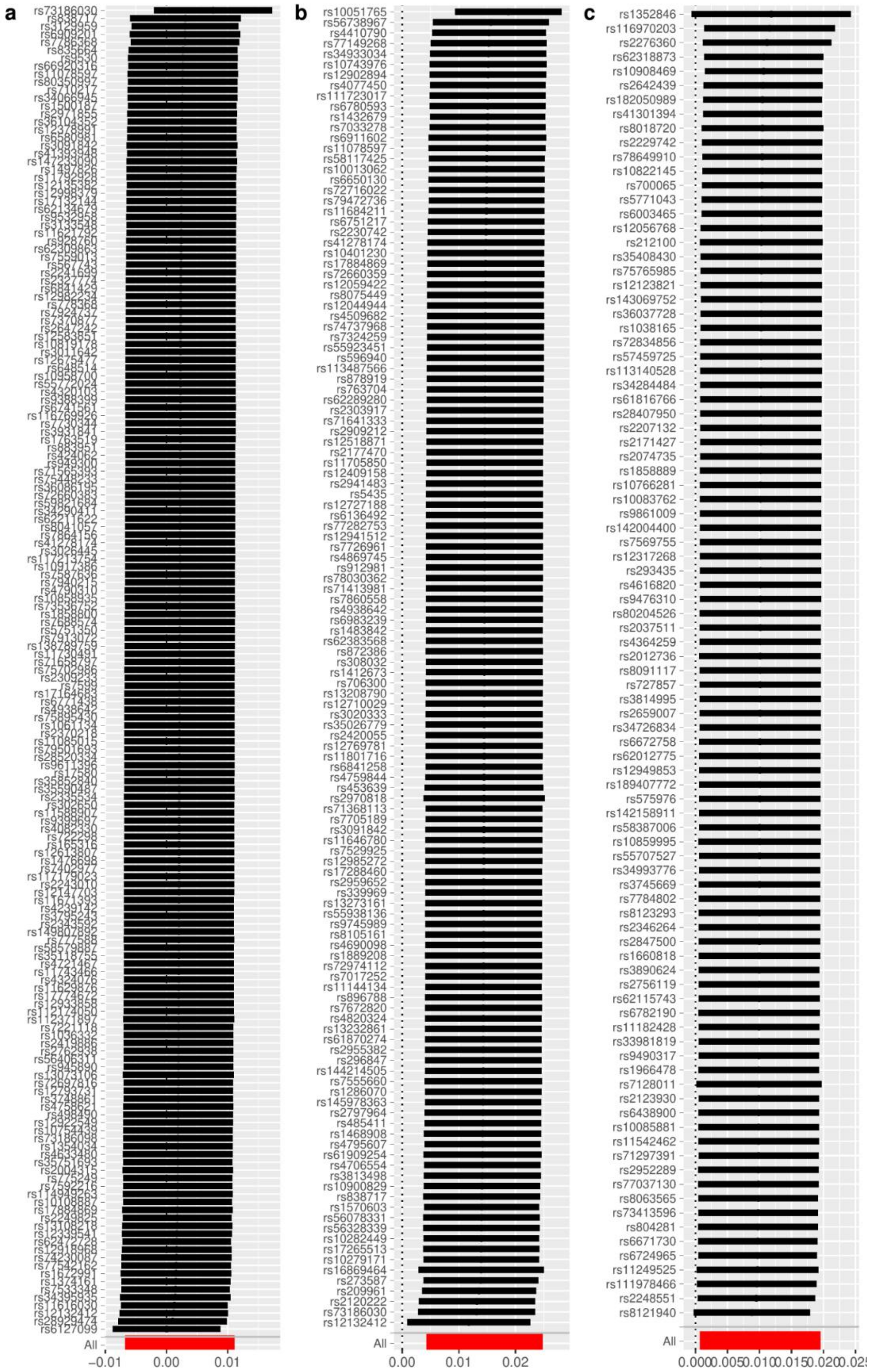

(a)Exposure: calcium; Outcome: BUN; (b) Exposure: phosphate; Outcome: BUN; (c) Exposure: 25-hydroxyvitamin D; Outcome: BUN. BUN, blood urea nitrogen.

Figure S10. Plots of leave-one-out analyses of the relationship between each exposure factor and BUN using finally selected instrumental variables.

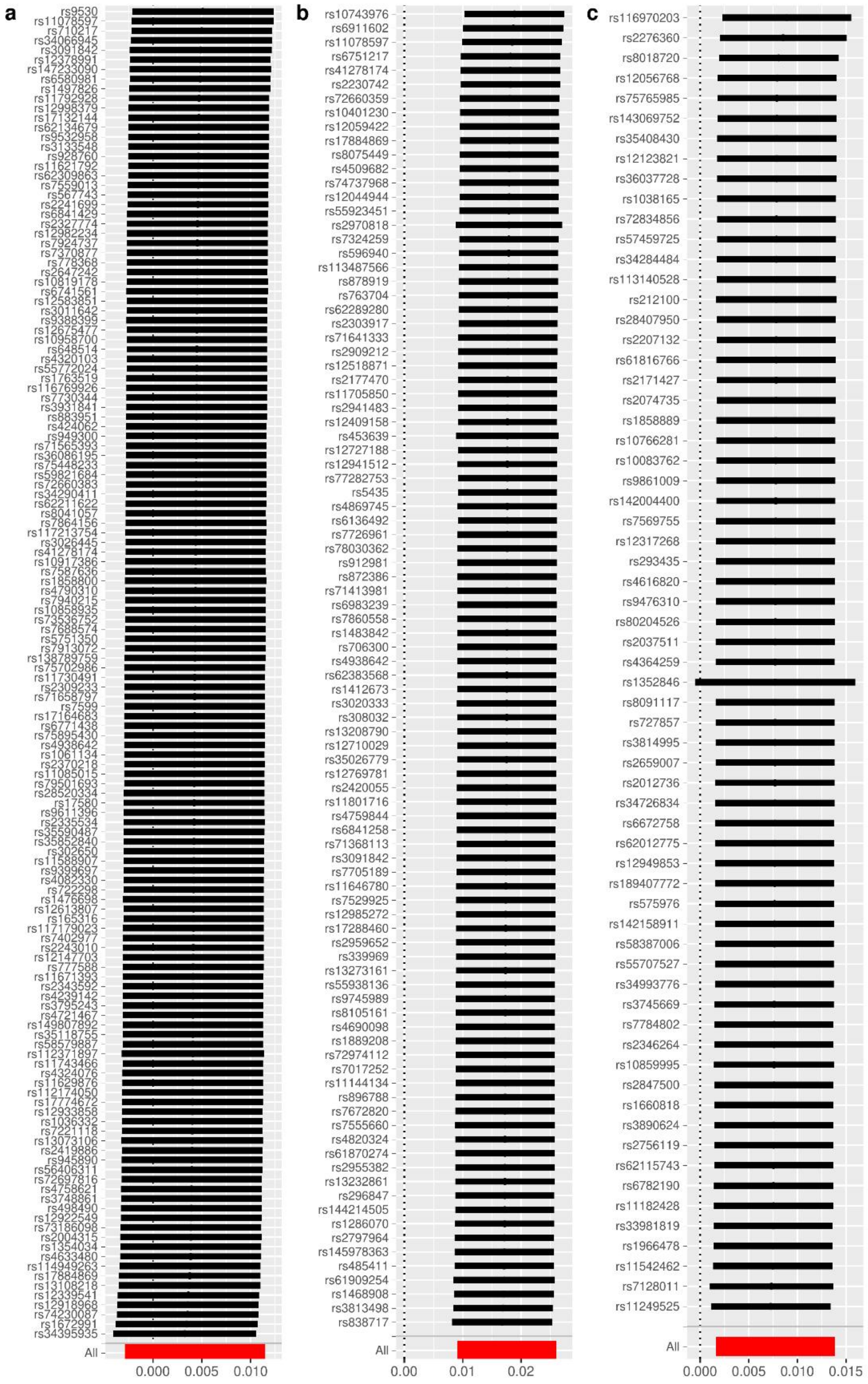

(a)Exposure: calcium; Outcome: BUN; (b) Exposure: phosphate; Outcome: BUN; (c) Exposure: 25-hydroxyvitamin D; Outcome: BUN. BUN, blood urea nitrogen.

Figure S11. Plots of leave-one-out analyses of the relationship between each exposure factor and UACR using initially selected instrumental variables.

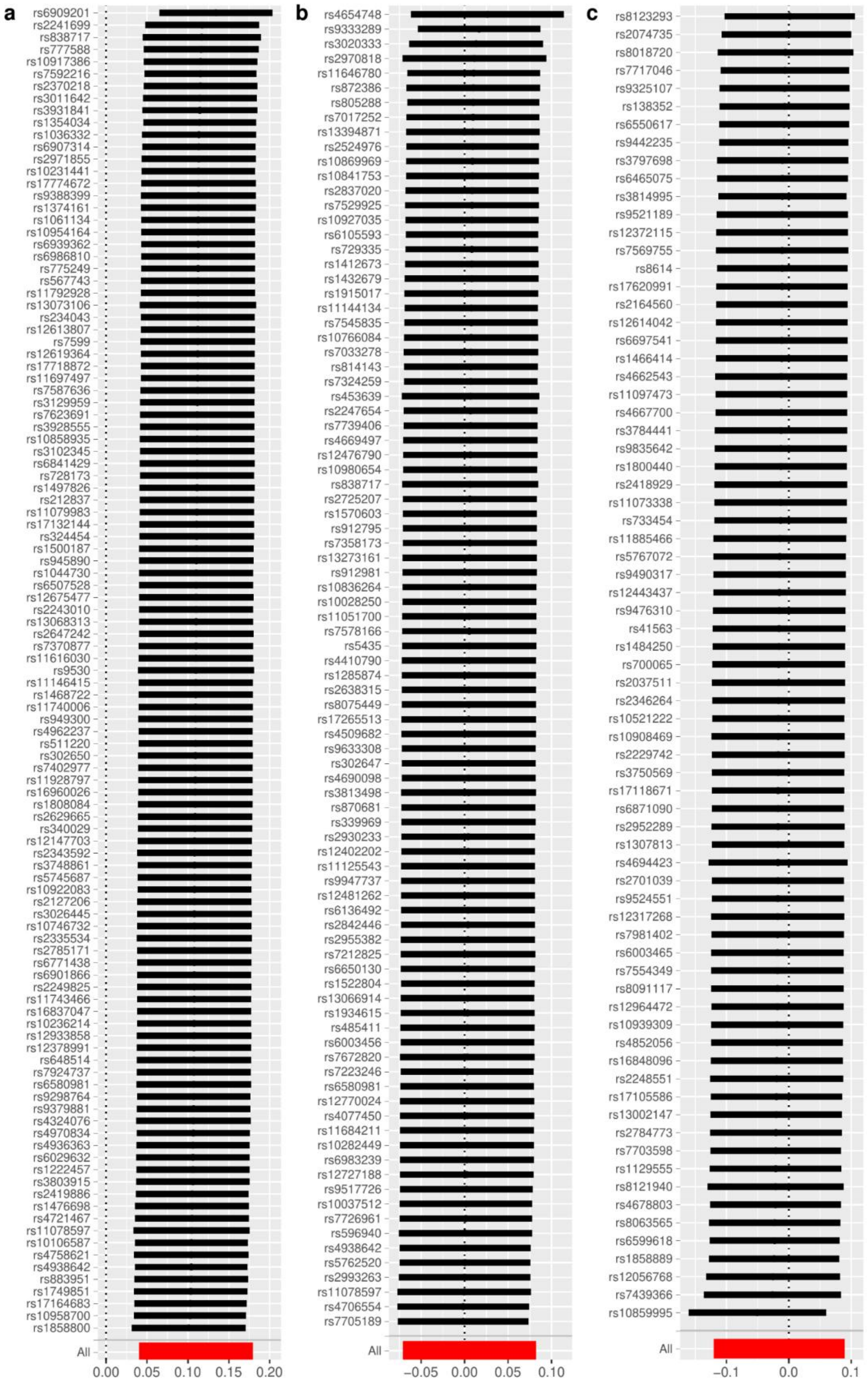

(a)Exposure: calcium; Outcome: UACR; (b) Exposure: phosphate; Outcome: UACR; (c) Exposure: 25-hydroxyvitamin D; Outcome: UACR. UACR, urinary albumin-creatinine ratio.

Figure S12. Plots of leave-one-out analyses of the relationship between each exposure factor and UACR using finally selected instrumental variables.

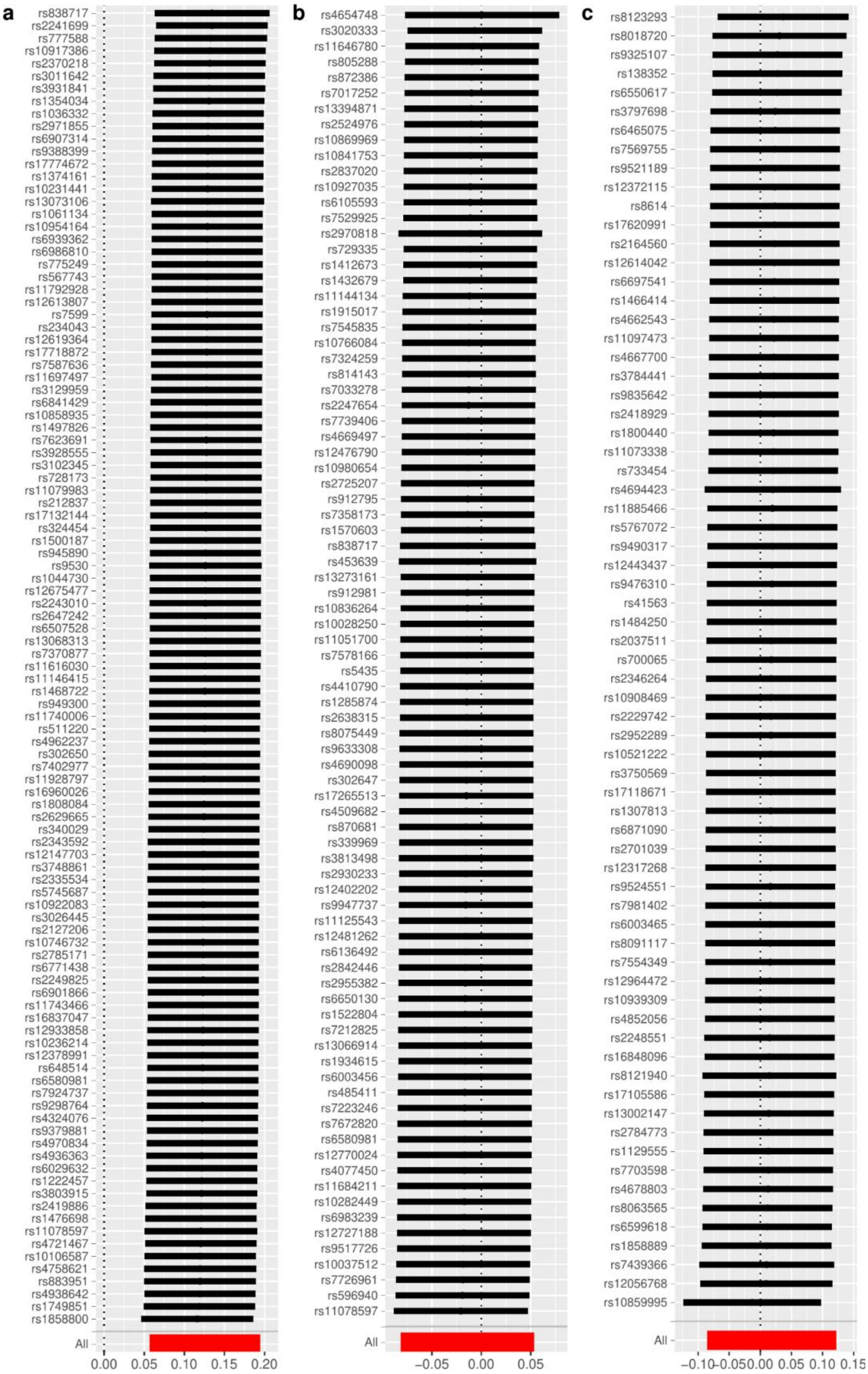

(a)Exposure: calcium; Outcome: UACR; (b) Exposure: phosphate; Outcome: UACR; (c) Exposure: 25-hydroxyvitamin D; Outcome: UACR. UACR, urinary albumin-creatinine ratio.
